# Low-temperature growth of layered molybdenum disulphide with controlled clusters

**DOI:** 10.1038/srep21854

**Published:** 2016-02-23

**Authors:** Jihun Mun, Yeongseok Kim, Il-Suk Kang, Sung Kyu Lim, Sang Jun Lee, Jeong Won Kim, Hyun Min Park, Taesung Kim, Sang-Woo Kang

**Affiliations:** 1 School of Mechanical Engineering, Sungkyunkwan University, Suwon, Gyeonggi, 440–746, Korea; 2 Center for Vacuum Technology, Korea Research Institute of Standards and Science, Daejeon 305–340, Korea; 3 National Nanofab Center, Korea Advanced Institute of Science and Technology, Daejeon 305–701, Korea; 4 Materials Genome Center, Korea Research Institute of Standards and Science, Daejeon 305–340, Korea; 5 SKKU Advanced Institute of Nanotechnology, Sungkyunkwan University, Suwon, Gyeonggi, 440–746, Korea; 6 Department of Advanced Device Technology, University of Science and Technology, Daejeon 305–350, Korea

## Abstract

Layered molybdenum disulphide was grown at a low-temperature of 350 °C using chemical vapour deposition by elaborately controlling the cluster size. The molybdenum disulphide grown under various sulphur-reaction-gas to molybdenum-precursor partial-pressure ratios were examined. Using spectroscopy and microscopy, the effect of the cluster size on the layered growth was investigated in terms of the morphology, grain size, and impurity incorporation. Triangular single-crystal domains were grown at an optimized sulphur-reaction-gas to molybdenum-precursor partial-pressure ratio. Furthermore, it is proved that the nucleation sites on the silicon-dioxide substrate were related with the grain size. A polycrystalline monolayer with the 100-nm grain size was grown on a nucleation site confined substrate by high-vacuum annealing. In addition, a field-effect transistor was fabricated with a MoS_2_ monolayer and exhibited a mobility and on/off ratio of 0.15 cm^2^ V^−1^ s^−1^ and 10^5^, respectively.

Diverse research has shown that graphene is a promising candidate for analogues of conventional electronic devices^1^,^2^,^3^. Although it possesses the extraordinary properties of a high electron mobility, elasticity, heat conductivity, and flexibility, graphene is not suitable for transistor and photonic devices owing to the lack of a bandgap (0 eV for pristine graphene). Molybdenum disulphide (MoS_2_), a layered structural material which coheres by the covalent bonding of one molybdenum atom between two sulphur atoms and interlayer van der Waals forces, has emerged as a new two-dimensional (2D) material owing to its tuneable band gap [from an indirect bandgap of 1.2 eV (bulk) to a direct bandgap of 1.8 eV (monolayer)]^4^ and ambient stability^5^.

The fabrication of a MoS_2_ monolayer was first attempted by a micromechanical exfoliation method similar to the approach used for the fabrication of graphene, and the possibility of using MoS_2_ as a channel material for a field-effect transistor (FET) was verified^6^,^7^. Recent achievements^8^ in obtaining high-performance FET devices using MoS_2_ as a channel material with a dielectric screening method^9^,^10^ gave rise to a number of synthetic processes such as micromechanical^11^,^12^,^13^,^14^ and chemical exfoiliation^15^,^16^, lithiation^17^, thermolysis^18^,^19^, and two-step thermal evaporation^20^. Subsequently, the sulphurization of a pre-deposited Mo^21^,^22^ was developed, and it was shown that sulphurization is a somewhat suitable method for the synthesis of large-area MoS_2_. However, MoS_2_ fabricated by the sulphurization of a pre-deposited Mo exhibit non-uniformity and a low field-effect mobility^21^ compared to exfoliated samples, and they occasionally grow perpendicular to the substrate^22^ because of ineffective incorporation of sulphur into the pre-deposited Mo. Chemical vapour deposition (CVD) is a well-known method for growing large-area MoS_2_. Lee *et al.* (ref. 23) demonstrated that CVD using molybdenum oxisulphides (MoO_3−x_) reduced from molybdenum trioxide (MoO_3_) and sulphur powder is a highly effective method for growing MoS_2_ atomic layers on a dielectric substrate. Studies^24^,^25^,^26^,^27^,^28^,^29^,^30^,^31^,^32^ with a similar method have demonstrated the effective growth of large-area^24^, high-quality MoS_2_ with a larger grain size^25^,^26^,^27^ and control of the number of layers^31^.

However, to the best of our knowledge, a feasible method for growing a MoS_2_ at low-temperatures of below 400 °C has not yet been reported, as it still requires the sulphurization of MoO_3−x_ at high temperatures ranging from 650 to 850 °C. The 2D materials are suitable materials for next-generation electronic devices such as flexible, stretchable, and wearable devices. This devices normally fabricated based on plastic substrate. However, the melting temperature of most plastic substrates (PET, PEN, PI, etc.) are lower than 400 °C that makes impossible to use high-temperature approaches for direct growth. The conventional method to fabricate the flexible devices is using transfer of high-temperature grown 2D materials to plastic substrates^33^. This transfer method does not guarantee the productivity and reproducibility compared to direct growth owing to structure deformation (cracks and wrinkles)^34^,^35^ and remained polymer residues^36^. Thus, low-temperature growth method can open the cost- and time- effective method for fabrication of flexible devices. Notwithstanding the few works which demonstrated the possible methods for low-temperature growth^37^,^38^,^39^, the electrical performance of as-grown MoS_2_ were not reported but only the processes and characterization of samples were presented. Typically, higher temperatures facilitate the growth of a high-quality film owing to the small number of nuclei, the long diffusion length on the surface, and the effective desorption of volatile substances. However, at lower temperatures, the growth of high-quality films is challenging, especially for monolayer growth owing to the small critical radius for nucleation and the short diffusion length on the surface. Herein, we report a direct one-step low-temperature CVD process for the growth of high-quality layered MoS_2_ with control of the cluster size and the nucleation sites using Mo(CO)_6_ and hydrogen sulphide (H_2_S) as the precursor and reaction gas, respectively. Spectroscopic and microscopic analyses demonstrate that differently structured (3D or 2D) MoS_2_ are formed by changing the S-reaction-gas (P_SR_) to Mo-precursor (P_MoP_) partial-pressure ratio (P_SR_/P_MoP_), and monolayer islands of MoS_2_ with a grain size of 100 nm were grown on a nucleation-site-confined silicon dioxide (SiO_2_) substrate with an optimized P_SR_/P_MoP_. In addition, the electrical performance of back-gate FET device using monolayer MoS_2_ was examined.

## Results and Discussion

**Figure 1:**
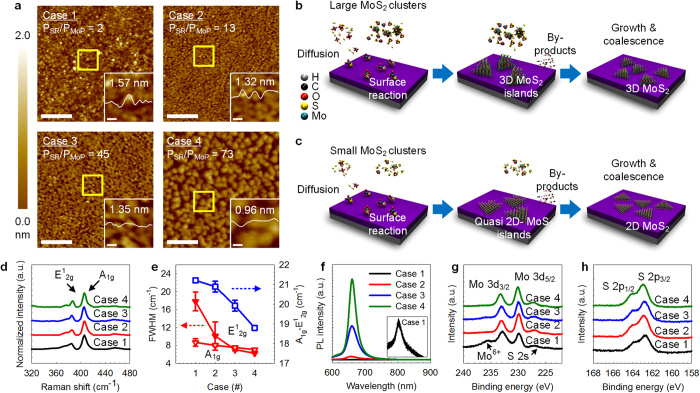
MoS_2_ with different structures and their growth mechanisms. (**a**) AFM images of MoS_2_ with different structures (3D: cases 1 and 2, 3D+2D: case 3, 2D: case 4) grown at various values of P_SR_/P_MoP_. The scale bar is 200 nm. The measured height profiles of the islands are shown in the inset figures (scale bar: 20 nm) indicated by the open yellow rectangles. (**b,c**) Illustration of our cluster-size control mechanism. Larger MoS_2_ clusters were formed by a gas-phase reaction at a lower P_SR_/P_MoP_ (**b**) whereas the formation of clusters was limited at a higher P_SR_/P_MoP_ (**c**). (**d,e**) Corresponding Raman spectra of each sample. The values of ∆k decreased from 21.7 to 18.8 cm^−1^ at P_SR_/P_MoP_ = 73 (**d**). The FWHMs of the two dominant modes decreased from 17.84 to 6.27 cm^−1^ (E^1^_2g_) and 8.68 to 6.75 cm^−1^ (A_1g_) (**e**). Silicon peak (520.8 cm^−1^) used for normalization. (**f**) Photoluminescence spectra of each sample. A higher intensity indicates that high-quality MoS_2_ was grown. (**g,h**) XPS spectra of each sample. The presence of Mo^6+^ in case 1 shows that oxides are incorporated with Mo.

It is known that a CVD process using Mo(CO)_6_ tend to create large aggregates^40^, Mo-based 3D structured films^41^,^42^ and films containing considerable amounts of carbides or oxides, such as Mo_2_C or MoOC, depending on the deposition conditions^43^. Although many disadvantages are caused by the carbonyl (CO) ligand radiating from the central Mo atom, lower decomposition temperature (Supplementary Fig. S1) make Mo(CO)_6_ a suitable precursor for low-temperature growth. To achieve the 2D growth of layered MoS_2_ at 350 °C, we developed a novel method that control the cluster size by feeding precise amount of Mo precursor and the nucleation sites on the SiO_2_ substrate by high-vacuum annealing. Although previous studies have verified that large amounts of carrier gas (Ar or H_2_) can facilitate decarbonylation^38^, the use of carrier gases is excluded in our experiment because large amounts of carrier gas eventually increase the absolute amount of precursor vapour. In order to examine our strategic approach, the experiment was carried out under various P_SR_/P_MoP_. The P_MoP_ was precisely controlled using a chiller–heater unit connected to a precursor canister (Supplementary Fig. S2a). Growth was carried out using a showerhead-type reactor to assist in the creation of a uniform flow^32^ (Supplementary Fig. S2b). Before growth, the SiO_2_ substrate was pre-cleaned using acetone, isopropyl alcohol (IPA), and deionized (DI) water to prevent nucleation near dust particles^26^. Subsequently, the substrate was loaded into a load-lock chamber for several seconds to prevent any surface contamination under ambient conditions and transported to the main reactor followed by growth for a specific time at a substrate temperature of 350 °C at various Mo(CO)_6_ sublimation temperatures (0 to 80 °C) and H_2_S flow rates (10 to 100 sccm). In our preliminary experiment conducted with a lower P_SR_/P_MoP_, structural changes and impurities incorporation (Supplementary Fig. S3) in MoS_2_ depending on the cluster size were observed and revealed that the partial-pressure ratio is the key parameter for 2D growth (Supplementary Fig. S3a). Figure 1a shows atomic force microscopy (AFM) images of various samples grown at different values of P_SR_/P_MoP_. At a lower P_SR_/P_MoP_ (case 1 and 2), irregular 3D islands with small grain sizes were grown. As P_SR_/P_MoP_ (case 3) increases, the morphology was changed to a mixed structure which was consisted of irregular 3D islands and 2D triangular islands. At a much higher P_SR_/P_MoP_, the structure completely changed to 2D triangular islands with larger grain sizes (case 4). The fact that 3D structural MoS_2_ formation at lower P_SR_/P_MoP_ compare to higher P_SR_/P_MoP_ may affected by cluster formation in gas phase. At a lower P_SR_/P_MoP_, a larger amount of Mo(CO)_6_ vapour sublimes and larger-size MoS_2_ clusters are formed by the gas-phase reactions. Consequently, the formed clusters were adsorbed onto the surface, and 3D MoS_2_ islands were grown (Fig. 1b). At a higher P_SR_/P_MoP_, quasi-2D MoS_2_ islands are grown on the surface by desorbing volatile by-products and transformed into monolayer MoS_2_ by surface diffusion (Fig. 1c). The Raman spectroscopy results of the grown MoS_2_ are in agreement with the corresponding atomic structure measurement results (Fig. 1d). The difference between two Raman modes (∆k) resulting from in-plane vibration (E^1^_2g_) and out-of-plane vibration (A_1g_) was measured 21.7 cm^−1^ for MoS_2_ grown at a lower P_SR_/P_MoP_ (cases 1–3) owing to the coincidence of monolayer and bilayer MoS_2_ (Fig. 1d), and it further decreased to 18.8 cm^−1^ at a higher P_SR_/P_MoP_ (case 4) with a decrease in the full width at half maximum (FWHM) of the E^1^_2g_ mode (Fig. 1e) and an increase in the photoluminescence (Fig. 1f). The FWHM of the PL spectra at higher P_SR_/P_MoP_ (case 4, Fig. 1f) measured as 26.3 nm, in comparable with high-temperature grown MoS_2_^27^. These results indicate that 2D structural MoS_2_ could be grown at under the higher P_SR_/P_MoP_ condition. The formation of 2D islands is elucidated by a theoretical consideration of the chemical potential and surface energy. Schweiger *et al.* (ref. 44) revealed that the type of edge termination (Mo- or S-edge) and the coverage by sulphur atoms of the monolayer MoS_2_ cluster were affected by the chemical potential of sulphur and the relationship with the corresponding parameters such as the ratio of S to Mo (Supplementary Fig. S4). Under strongly sulphiding conditions (high H_2_S partial pressure), the lower chemical potential of sulphur causes 100% coverage of the Mo edge (or S edge) by 100% sulphur to have the lowest surface energy. Under these conditions, the layer atoms are more strongly attracted to the substrate than to themselves, thereby facilitating 2D growth. The S-to-Mo ratios of 1.37, 1.99, 1.95, and 2.27 were measured for MoS_2_ grown from lower to higher values of P_SR_/P_MoP_ from X-ray photoelectron spectroscopic (XPS) analyses (Fig. 1g,h). These observations explain the structural changes and demonstrate that control of cluster size and strongly sulphiding conditions are a crucial factor for the layered growth of a MoS_2_ at lower temperatures.

**Figure 2:**
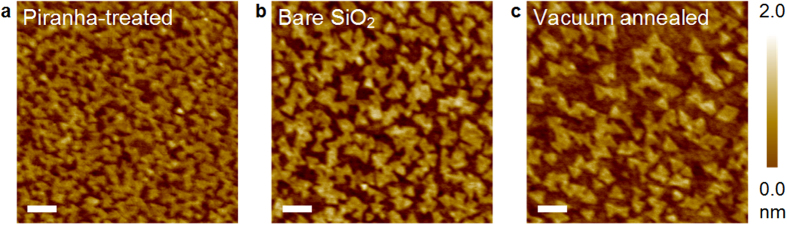
Nucleation site effect. **(a–c**) AFM images of MoS_2_ monolayer islands grown on different substrates: (**a**) piranha-treated, (**b**) bare, and (**c**) high-vacuum annealed. The piranha treatment passivates the dangling bonds, whereas the high-vacuum annealing de-passivates the passivated dangling bonds in bare SiO_2_. Larger-size islands were grown on the high-vacuum annealed SiO_2_ substrate owing to the confined nucleation site. The growth time is 12 h and P_SR_/P_MoP_ = 314. The scale bar is 100 nm.

The grain size of polycrystalline 2D materials is the most important characteristic for determining its physical and electrical properties^45^. At lower temperatures, the grain size of 2D materials is much smaller than those at higher temperatures owing to the small diffusion length on the surface. We observed single-crystal monolayer MoS_2_ domains grown at various values of P_SR_/P_MoP_ by AFM (Supplementary Fig. S5). However, no grain sizes greater than 50 nm were observed under even strongly sulphiding conditions (P_SR_/P_MoP_ = 594). This experiment reveals the existence of a grain-size limit at 350 °C. To overcome this limitation due to the short diffusion length on the surface, nucleation sites were artificially manipulated by annealing the substrate in high-vacuum. To examine the effect of nucleation-site manipulation on the grain size, we grew monolayer MoS_2_ on three different substrates: piranha (H_2_SO_4_:H_2_O_2_ = 3:1)-treated, bare, and high-vacuum annealed SiO_2_ substrates, as seen in Fig. 2. A larger number of triangular MoS_2_ islands with the smaller grain size was created on the piranha-treated substrate (Fig. 2a), whereas a smaller number of islands with the larger grain size was created on the vacuum-annealed substrate (Fig. 2c) compared to that of the bare SiO_2_ substrate (Fig. 2b). It is known that the hydroxylated or hydrogen-passivated dangling bonds of amorphous SiO_2_ provide many reactive surface sites compared to an unsaturated surface^46^,^47^. In contrast, the high-vacuum annealing treatment dissociates the hydrogen-passivated dangling-bond entities^47^,^48^. To clarify the nucleation and growth mechanism on the different substrates, the AFM images obtained at different growth times demonstrate that the MoS_2_ nuclei occupy every preferred nucleation site during the early phase of growth and then attach to the edges of as-grown monolayer islands, and no more nucleation was observed during growth (Supplementary Fig. S6). The monolayer MoS_2_ islands were grown up to 100 nm on the nucleation-site-confined substrate. For the growth of MoS_2_ with larger grain sizes at lower temperatures, it is crucial to manipulate the affinity of the nuclei and the substrate; thus, the grain-size limitation can be overcome. The effect of substrate temperature on grain size was also examined (Supplementary Fig. S7). The grain size and FWHM of E^1^_2g_ mode of grown MoS_2_ were decreased by decreasing temperature owing to short diffusion length on the surface.

**Figure 3:**
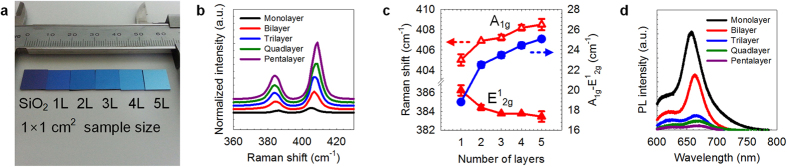
Layered MoS_2_. (**a**) Photograph of bare SiO_2_ and monolayer to pentalayer MoS_2_ grown onto a 1 × 1 cm^2^ SiO_2_ substrate. The layer is controlled with the growth time and no other conditions are changed. (**b,c**) Raman spectra of layered MoS_2_. The E^1^_2g_ and A_1g_ modes are red- and blue-shifted by increasing the number of layers, respectively. The values of ∆k were measured as 18.8, 22.6, 23.6, 24.5, and 25 cm^−1^ for monolayer to pentalayer MoS_2_. (**d**), Photoluminescence of layered MoS_2_. Two dominant absorption peaks (near 670 and 620 nm) corresponding to two direct excitonic transitions (A1 and B1) are observed, and their intensities decrease as the number of layers increases. The indirect bandgap transition is not observable in multi-layered samples, which is the usual phenomenon on a SiO_2_ substrate.

The number of a MoS_2_ layer has been conventionally controlled by modulating the thickness of the pre-deposited Mo^21^, the surface energy^31^, or the supersaturation^38^. The grown MoS_2_ using our method exhibit the characteristic of layered growth (the detailed growth process is shown in Supplementary Fig. S8) without changing other parameters. Different surface colours were observed for different numbers of layers in Fig. 3a, in which highly uniform large-area MoS_2_ were grown on 1 × 1 cm^2^ SiO_2_ substrates and grown at the wafer scale up to 3” in size (Supplementary Fig. S9), as confirmed by an ellipsometry mapping analysis. We also used Raman spectroscopy and photoluminescence measurements to confirm the thickness of the as-grown MoS_2_. The Raman spectrum of each sample exhibits red and blue shifts of the E^1^_2g_ and A_1g_, respectively, as the number of layers increases (Fig. 3b). The ∆k values were measured to be 18.8, 22.6, 23.6, 24.5, and 25 cm^−1^ (Fig. 3c) from monolayer to pentalayer^49^,^50^. The normalized intensity was increased for thicker MoS_2_ owing to optical interference effect on SiO_2_/Si^49^. When the substrate temperature decreased to 250 °C, the bilayer islands were grown on uncovered monolayer MoS_2_ owing to short diffusion length (Supplementary Fig. S7). The two dominant absorption peaks (near 670 and 620 nm) correspond to two direct excitonic transitions (A1 and B1, respectively) which were observed from the photoluminescence measurements. The intensity of A1 direct excitonic transition was decreased and shifted to the red with increasing number of layer (Supplementary Fig. S8i), in agreement with previous reports^4^,^5^. Our cluster-size control method provides a feasible way for the layered growth of MoS_2_ at the wafer scale and open the effective way for the photoelectric device applications without transfer process.

**Figure 4:**
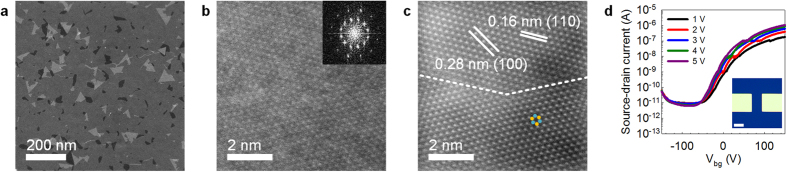
Atomic structures and electrical performance. **(a**) Low-magnification STEM-HAADF image of polycrystalline monolayer MoS_2_. Triangular single domains with an approximate size of 100 nm can be observed and create grain boundaries. (**b**) High-magnification STEM-HAADF image of a grain boundary. Two adjacent single-crystal domains create a grain boundary with a 31° tilt angle. The inset shows the FFT pattern which shows the hexagonal structure of the MoS_2_ monolayer. (**c**), Smoothed and Fourier-filtered image of Fig. 4b. Highly uniform and defect-free structures are observed with brighter Mo atoms and darker S atoms. (**d**) The electrical characteristics of the fabricated FET devices with a 5 μm and 10 μm channel length and width (Inset, scale bar: 5 μm). A mobility of 0.15 cm^2^V^−1^s^−1^ and a maximum on/off ratio of 10^5^ at 5 V are measured with an applied back-gate voltage ranging from −150 to 150 V and a bias voltage from 1 to 5 V. The monolayer MoS_2_ was not patterned.

The atomic structure of an as-grown monolayer MoS_2_ was evaluated by aberration-corrected scanning transmission electron microscopy (Cs-STEM) high-angle annular dark-field (HAADF) imaging. Figure 4a shows a low-magnification STEM-HAADF image of a MoS_2_ monolayer transferred onto a carbon grid by a conventional wet-etching method. The white region represents the overlapping MoS_2_ monolayer during transfer, and the grey region indicates a polycrystalline MoS_2_ monolayer. The approximate domain size is 100 nm and is in agreement with our previous observations using AFM (Supplementary Fig. S6b). The high-magnification HAADF image of the selected area shows the atomic structure of the grain boundary by two triangular domains (Fig. 4b). The fast Fourier transform (FFT) patterns in the inset of Fig. 4b indicate the hexagonal structures of the two single-crystal MoS_2_ domains with a 31° tilt angle. From the image reconstructed by smoothing and Fourier filtering (Fig. 4c), a uniform single-crystal MoS_2_ domain was observed, and the merge to create a grain boundary (indicated by the dashed white line in Fig. 4c), thereby forming a polycrystalline MoS_2_ monolayer. Moreover, the samples grown at a higher P_SR_/P_MoP_ exhibit better quality compared to those grown at a lower P_SR_/P_MoP_ (Supplementary Fig. S10). This microscopic observation reveals that a highly uniform and large-grain MoS_2_ polycrystalline monolayer was grown even at 350 °C. Furthermore, the domain structure and grain boundary closely resemble MoS_2_ grown at higher temperature. The low-temperature grown monolayer MoS_2_ was used to fabricate a back-gate FET to examine the electrical performance. The device was fabricated with a MoS_2_ monolayer without patterning and has a channel length and width of 5 and 10 μm, respectively (inset in Fig. 4d). The MoS_2_ monolayer was not treated after growth, and measurements were obtained at room temperature under ambient conditions. The FET device exhibits conventional n-type semiconductor behaviour with a mobility of 0.15 cm^2^V^−1^s^−1^ (Fig. 4d). The maximum on/off ratio was 10^5^ in the gate-voltage range of −150 to 150 V with a 5-V source–drain bias voltage that was ten times lower than high-temperature grown MoS_2_ by using CVD^24^,^25^,^26^,^27^ and exfoliated MoS_2_ (1–10 cm^2^V^−1^s^−1^).

## Conclusion

In conclusion, we developed a novel method for the layered growth of large area and high-quality MoS_2_ compare to other low-temperature method at a low-temperature of 350 °C using Mo(CO)_6_ by controlling the cluster size and nucleation sites. Furthermore, we first demonstrate the potential use of low-temperature grown MoS_2_ as practical FET device. A structural transition from 3D clusters to 2D monolayers by changing P_SR_/P_MoP_ and controlling the grain size with confined nucleation sites were demonstrated. These two parameters are key factors for the low-temperature growth of the layered MoS_2_. The low-temperature growth of 2D materials represented by graphene and transition-metal dichalcogenides is crucial for the application of next-generation flexible and wearable devices. Thus, our results suggest novel approaches for the preparation of 2D materials under lower temperature conditions.

## Methods

### Growth process

Layered MoS_2_ was grown by a showerhead-type reactor using Mo(CO)_6_ (≥99.9%, Sigma Aldrich, CAS number 13939-06-5) as a precursor. Highly doped (<0.005 Ω·cm) p-type Si with a 300-nm-thick SiO_2_ layer was used as the substrate. The substrates were pre-cleaned and placed onto a silicon carbide (SiC)-coated susceptor in a load-lock chamber within a short period to prevent any contamination in the ambient environment. The heating block in the CVD reactor was pre-heated to 350 °C before growth. The susceptor with the substrate was transferred to the reactor, and the substrate temperature was increased over a period of 10 min in an Ar flow having a purity of 99.999%. The growth was carried out using only sublimed precursor vapour with a high-purity H_2_S flow for growth times at a constant pressure of 0.5 Torr. The substrates were transferred to the load-lock chamber after growth and cooled down for 1 h with 100 sccm Ar flow (Supplementary Fig. S2a). The treatment after growth was not carried out with any known method (such as Ar and H_2_S annealing at a high temperature). All analyses and characterization were performed using as-grown samples.

### AFM measurement

The morphology, grain size, and nucleation and growth processes were evaluated using AFM (XE-150, Park Systems). For better quality, an image was measured using a super sharp silicon tip with a radius of curvature of <5 nm (SSS-NCHR, NANOSENSORS). A soft X-ray ionizer module was applied to prevent electrostatic charge during measurement. The image was taken over a 1 or 2 μm^2^ area with a 512 × 512 pixel resolution and a measurement speed of 0.5 Hz. The images were resized to 750 nm^2^.

### Spectroscopy

Raman spectroscopy measurements were carried out using a DXR Raman Microscope (Thermo Scientific). A laser with an excitation wavelength of 532 nm, a spot size of 0.7 μm, and a power of 8 mW was used. The approximate spectral resolution is 0.5 cm^−1^, and the 520.8 cm^−1^ Si peak was used for normalization. Photoluminescence (LabRam ARAMIS, Horiba Jobin Yvon) measurements of the grown samples were carried out with a wavelength of 514 nm and a laser power of 10 mW. The ellipsometry (M2000D, J. A. Woollam Co.) mapping measurements were carried out with a 0.5-cm step size. The thickness results were extracted by multi-layer (four-layer model, air/MoS_2_/SiO_2_/Si) modelling. XPS (SES-100, VG-SCIENTA) measurements were conducted using a non-monochromatic magnesium Kα source under ultra-high vacuum conditions (<10^−8 ^Torr).

### TEM sample preparation

Poly(methyl methacrylate) (PMMA) (950 A2, MicroChem) was spin-coated on as-grown MoS_2_/SiO_2_/Si samples at 4,000 rpm for 60 s. The SiO_2_ layer was etched away by immersing the coated samples in a buffered oxide etch (BOE) solution (6:1, J.T.Baker). The detached PMMA/MoS_2_ was rinsed several times with DI water and then simply placed onto carbon grids (HC300-CU, Electron Microscopy Sciences). PMMA was removed by annealing under high-vacuum conditions (<10^−5 ^Torr) at 300 °C for 30 min (see ref. 25).

### HAADF-STEM

HAADF-STEM images were taken using Cs-STEM (Titan cubed G2 60-300, FEI) operated at 300 kV with a 50–100 pA screen current and a 19.3 mrad convergence angle. The images were further smoothed and Fourier filtered to improve the contrast.

### Electrical performance measurement

The back-gate FET device was fabricated by using electron-beam evaporation to deposit Ti/Au (5/50 nm) electrodes directly onto an as-grown MoS_2_ monolayer. The electrode shapes were patterned using electron-beam lithography of a PMMA (950 C4, MicroChem) layer and developed with diluted MIBK (MIBK:IPA = 1:1, MicroChem) solution. The lift-off process was conducted by immersion into dichloromethane (DCM) and IPA and drying with high-purity N_2_ (99.999%). The electrical performance of the device was measured at room temperature under ambient conditions using an in-house four-probe station with a precision semiconductor parameter analyser (4156A, Hewlett-Packard). The device was not annealed.

## Additional Information

**How to cite this article**: Mun, J. *et al.* Low-temperature growth of layered molybdenum disulphide with controlled clusters. *Sci. Rep.* **6**, 21854; doi: 10.1038/srep21854 (2016).

## Supplementary Material

Supplementary Information
