# Supplementary material for: Low-temperature growth of layered molybdenum disulphide with controlled clusters
Source: Sci Rep. 2016 Feb 23;6:21854. doi: 10.1038/srep21854 (PMC4763177; doi:10.1038/srep21854)
Supplement: Supplementary Information [file srep21854-s1.pdf]

Low-temperature growth of layered molybdenum disulphide with controlled clusters

Jihun Mun[1,2], Yeongseok Kim[1,2], Il-Suk Kang[3], Sung Kyu Lim[3], Sang Jun Lee[4], Jeong Won Kim[4], Hyun Min Park[4], Taesung Kim[1,5*], and Sang-Woo Kang[2,6*]

[1]School of Mechanical Engineering, Sungkyunkwan University, Suwon, Gyeonggi, 440-746, Korea

[2]Center for Vacuum Technology, Korea Research Institute of Standards and Science, Daejeon 305-340, Korea

[3]National Nanofab Center, Korea Advanced Institute of Science and Technology, Daejeon 305-701, Korea

[4]Materials Genome Center, Korea Research Institute of Standards and Science, Daejeon 305-340, Korea

[5]SKKU Advanced Institute of Nanotechnology, Sungkyunkwan University, Suwon, Gyeonggi, 440-746, Korea

[6]Department of Advanced Device Technology, University of Science and Technology, Daejeon 305-350, Korea

*tkim@skku.edu, +82-31-290-7466

*swkang@kriss.re.kr, +82-42-868-5823

Supplementary Table S1. XPS data of the MoS_2 with different structures grown at various values of P_{SR}/P_{MoP}.

Case #	Mo 6+	Mo 4+	S 2p	S/Mo Stoichiometry
1	1494.24	3448.52	6781.20	1.37
2	500.63	5837.95	12636.10	1.99
3	613.14	4767.36	10513.73	1.95
4	370.08	7994.50	19019.64	2.27

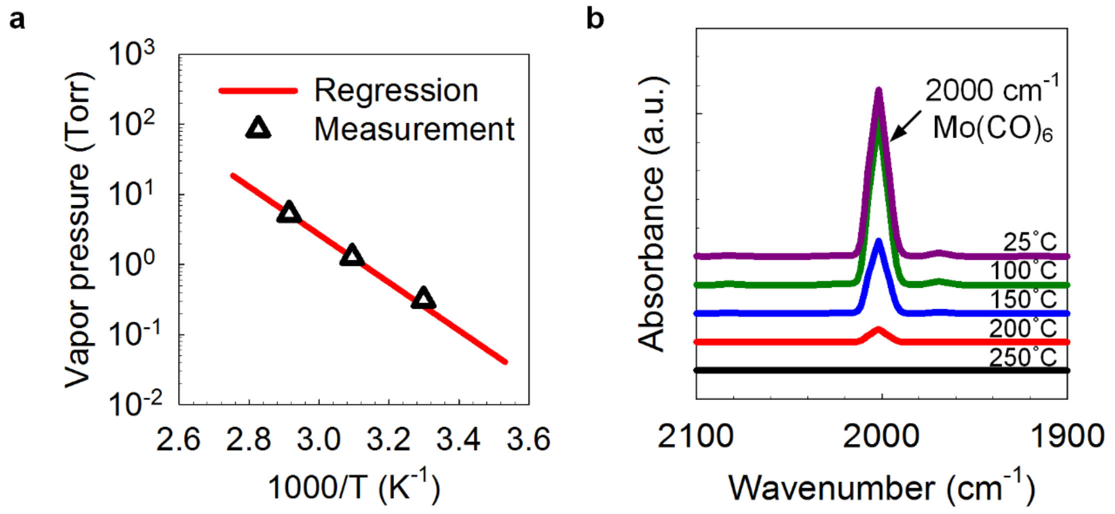

Supplementary Figure S1. Vapour pressure and decomposition characteristic measurement of $Mo(CO)_6$.

The vapour pressure and decomposition characteristic of the precursor is a crucial parameter when considering its suitability for the CVD process. For the vapour-pressure measurement, an in-house measurement system was used. A vessel with a specific volume was first maintained under high-vacuum conditions ($<10^5$ Torr) by a turbomolecular pump (TMP) followed by opening of a connected quartz tube containing the precursor and maintained for several hours until pressure remained constant. The vapour pressure of $Mo(CO)_6$ at precursor temperatures of 30, 50, and 70℃ were measured as 0.31, 1.27, and 5.24 Torr (Fig. S1a) and analysed using a simplified form of the Clapeyron equation[1,2], $\ln(P_{sat}/\mathrm{Pa}) = A - B/(T/K)$, where P_{sat} and T are the saturation vapour pressure and precursor temperature, respectively. The decomposition characteristic of $Mo(CO)_6$ was evaluated using FT-IR (Nicolet 6700, ThermoScientific). The primary absorption peak of $Mo(CO)_6$ was measured at 2000 cm^{-1}, which is in agreement with a previously reported work[3], and the precursor was fully decomposed at approximately 250℃ and a pressure of 0.5 Torr (Fig. S1b). The characteristics of a higher vapour pressure and lower decomposition temperature make $Mo(CO)_6$ a suitable precursor for the low-temperature CVD process.

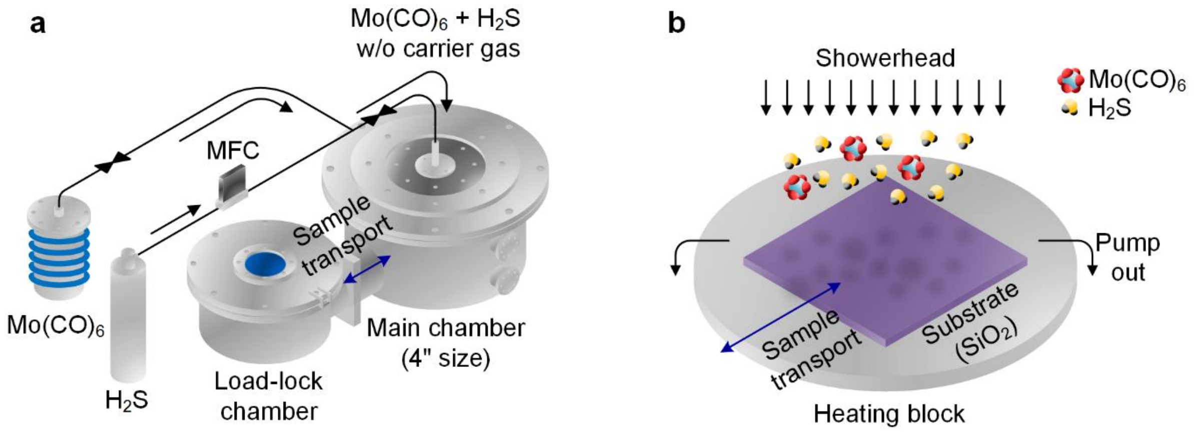

Supplementary Figure S2. CVD setup.

Low-temperature growth of layered MoS_2 was conducted using a showerhead-type reactor connected with a gas flow line and the load-lock chamber, as shown in Fig. S2a. The pre-cleaned or treated (by piranha or high-vacuum annealing) SiO_2 substrates were placed into the load-lock chamber followed by transfer of the sample to the main chamber. The partial pressure of $Mo(CO)_6$ was precisely controlled using a chiller–heater unit (the controllable range is -20 to 80℃). The flow rate of H_2S gas was controlled using a mass flow controller. The use of carrier gases (such as Ar and H_2) was excluded to prevent formation of larger size clusters.

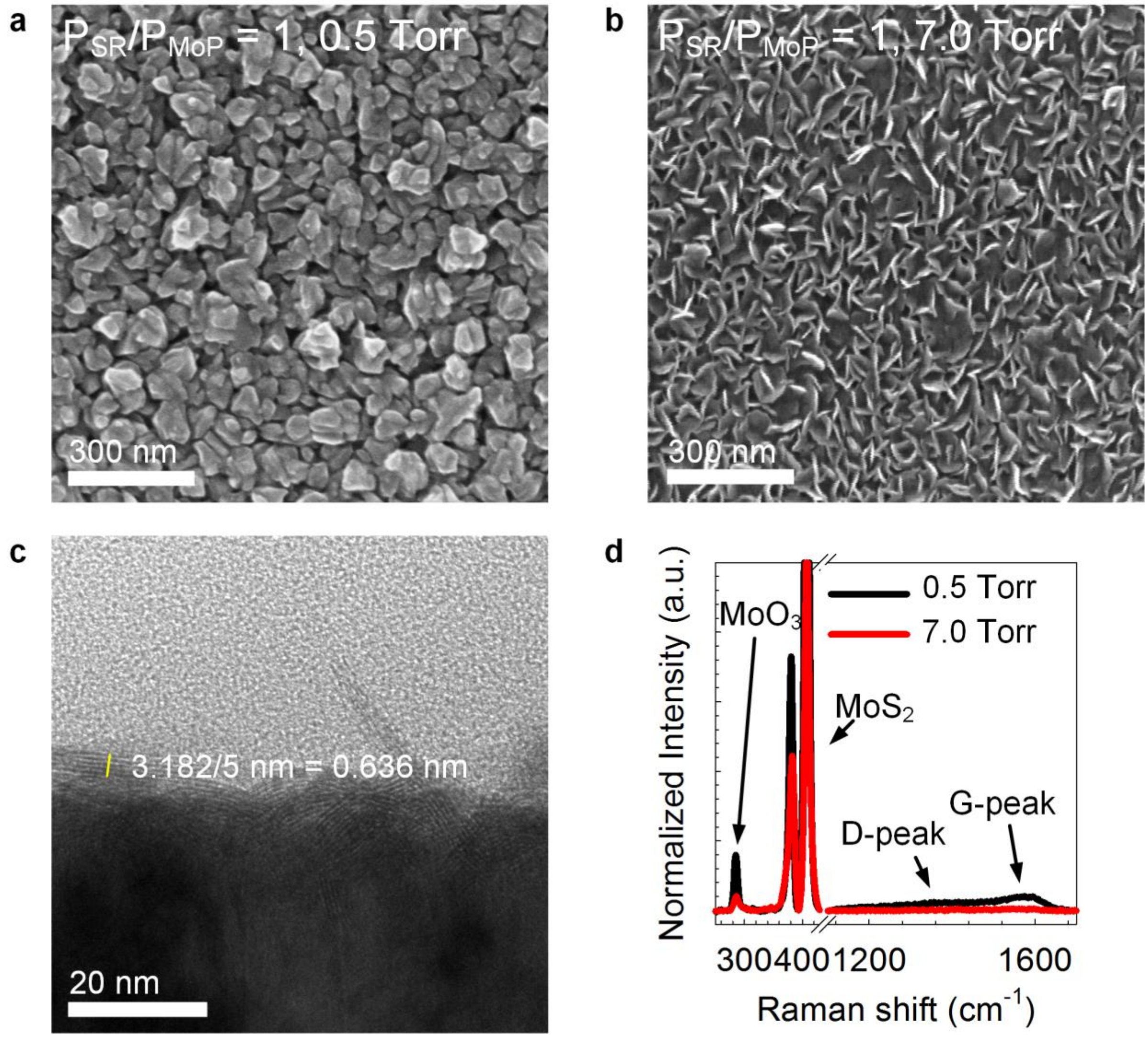

Supplementary Figure S3. 3D structural MoS_2.

We evaluate the MoS_2 grown at a lower P_{SR}/P_{MoP} using scanning electron microscopy (SEM, S-4800, HITACHI), high-resolution transmission electron microscopy (HRTEM, Tecnai G2 F30 S-Twin, FEI), and Raman spectroscopy. Larger size 3D structural MoS_2 was grown owing to the larger size clusters formed by the gas-phase reaction (Fig. S1a–c). MoS_2 incorporated with a larger amount of carbides and oxides (P_{SR}/P_{MoP}=1, 0.5 Torr) was grown and confirmed by Raman spectroscopy (285 cm^{-1} for the oxides, 1350 and 1580 cm^{-1} for the carbides).[4,5] The presence of carbides was not observed in MoS_2 grown under increased pressure conditions; only MoO_3 and MoS_2 peaks were measured. Although the partial

pressure of $Mo(CO)_6$ decreased as the chamber pressure increased, the partial pressure of H_2S did not. Consequently, P_{SR}/P_{MoP} increased, leading to the formation of smaller size clusters. This preliminary experiment shows the effect of the partial-pressure ratio on the structural change and feasible decarbonylation method, in which the cluster size is controlled.

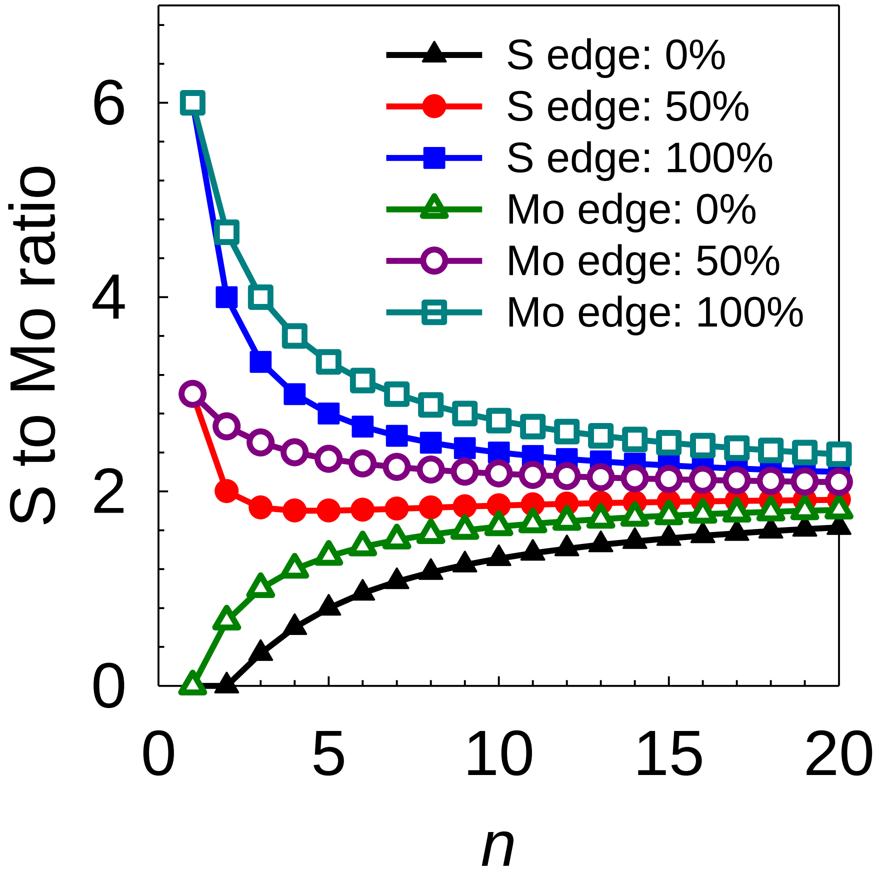

Supplementary Figure S4. MoS_2 edge type, sulphur coverage, and relevant S-to-Mo ratio.

It is known that two types of edge (Mo and S edge) structures can be formed under various conditions[6,7]. The created edge type and sulphur coverage with the relevant parameter of the S-to-Mo ratio elucidate the triangular monolayer domains which are often observed in high-quality MoS_2. Perfect, regular, triangularly shaped domains are present for the S-edge terminated 2D MoS_2 cluster covered with 100% sulphur atoms. This structure usually forms under highly sulphiding conditions. In Fig. S5, the calculated S-to-Mo ratio is plotted as a function of the cluster size *n* for various edge types and sulphur coverages[8,9]. The XPS measurement results and AFM images of MoS_2 grown at various values of P_{SR}/P_{MoP} reveal that a higher P_{SR}/P_{MoP} facilitates 2D growth by decreasing the surface energy.

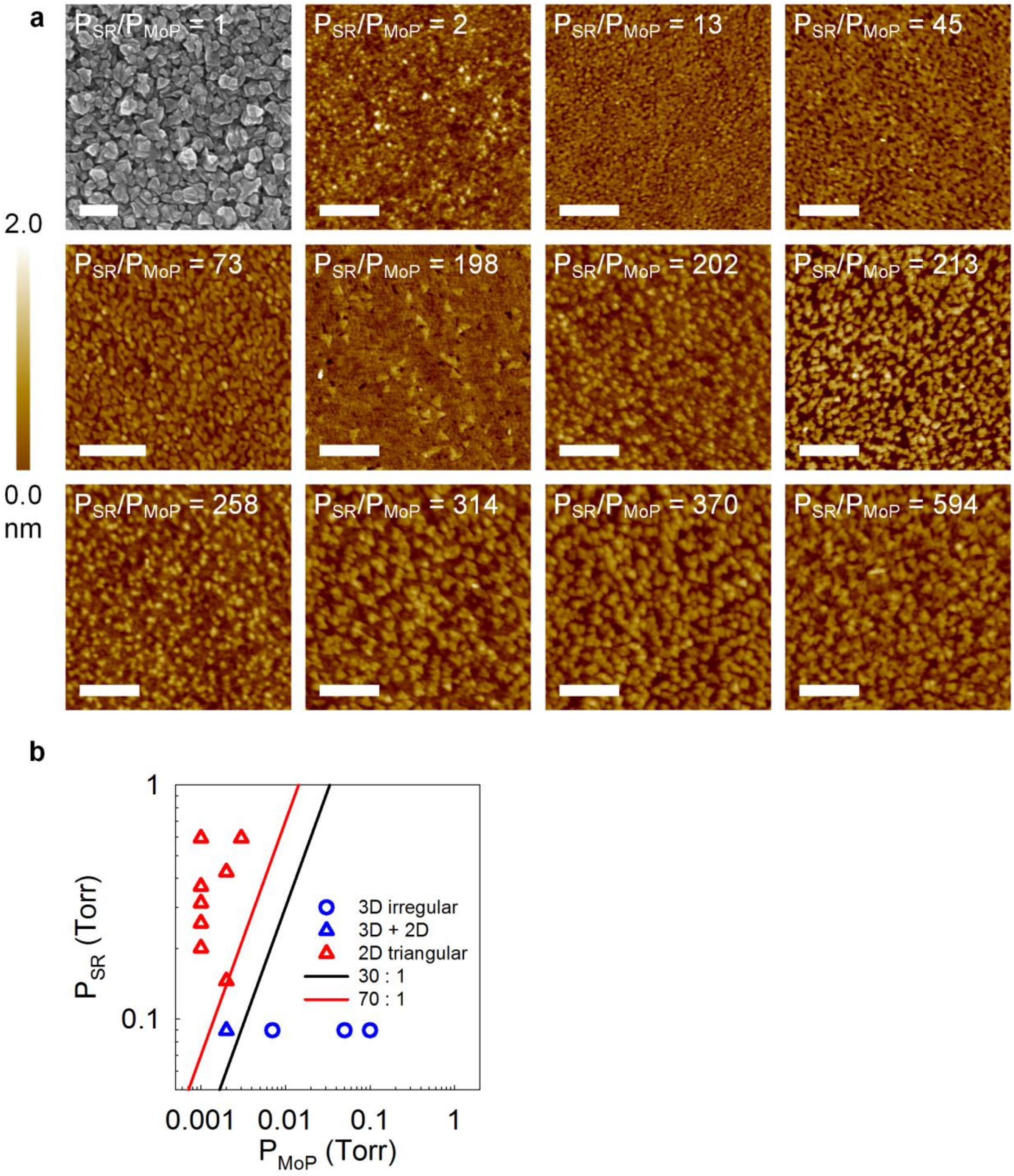

Supplementary Figure S5. MoS_2 grown at various values of P_{SR}/P_{MoP}.

Microscope images (Fig. S5a) for samples grown at various values of P_{SR}/P_{MoP} which show the growth window (Fig. S5b) at lower temperature. Triangular 2D MoS_2 islands were grown under at a specific P_{SR}/P_{MoP} of 73. The scale bar is 200 nm.

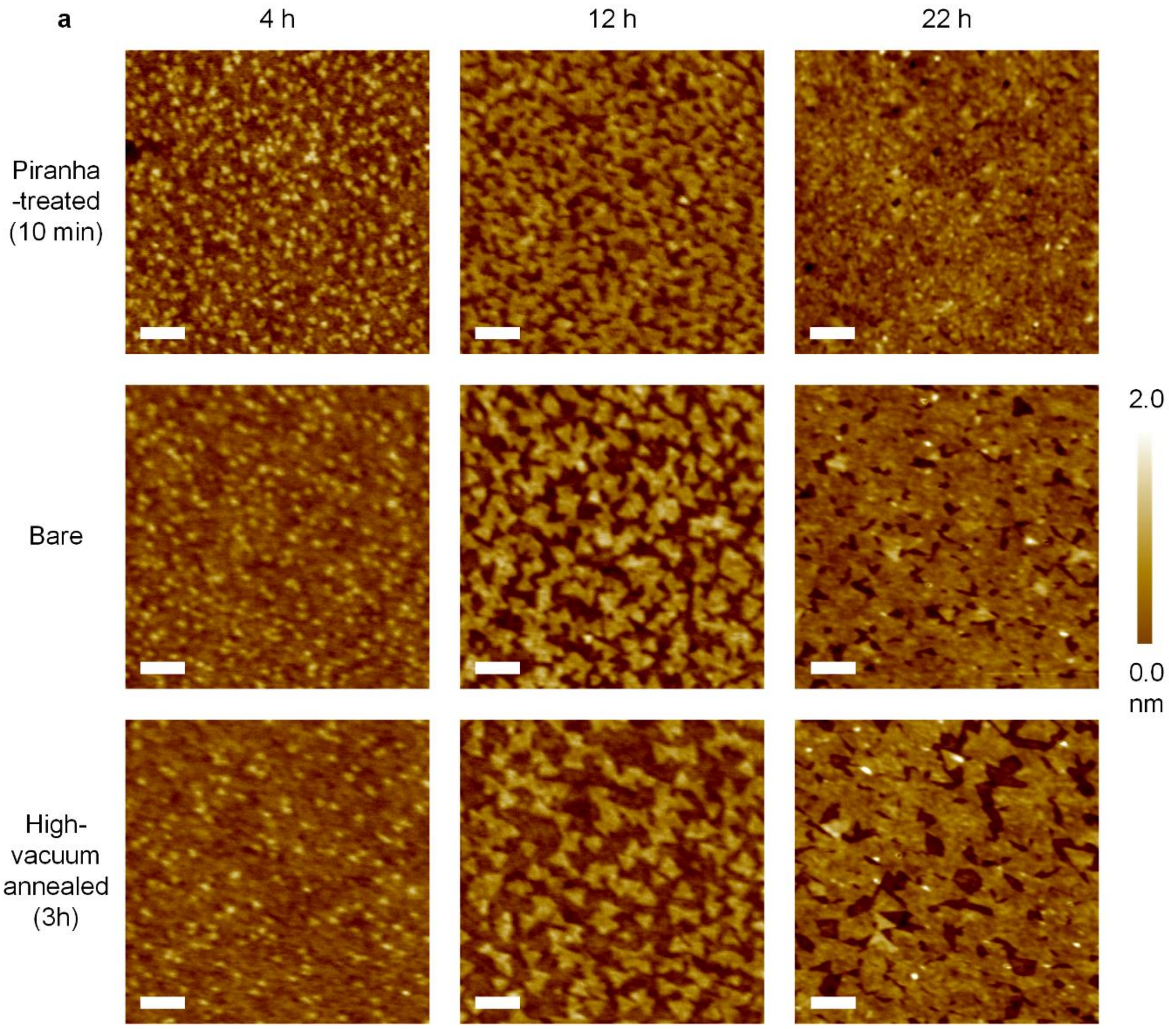

Supplementary Figure S6. MoS_2 grown for different growth times on different substrates.

The growth processes on different substrates for different growth times were observed using AFM (P_{SR}/P_{MoP}=146). For the treatment, bare SiO_2 was first cleaned with acetone, IPA, and DI water. Then, the substrate was immersed into the piranha solution for 10 min to hydroxylate the dangling bonds followed by rinsing with DI water. The bare SiO_2 substrate was annealed under high-vacuum conditions (<10^{-5} Torr) for 140 min at 750℃ to de-passivate the hydrogen-passivated dangling bonds[10]. A larger number of MoS_2 nuclei was observed for the nucleation sites on the piranha-treated substrate compared to the other two substrates, and new nucleation sites are not created during growth. Instead, the MoS_2 nuclei

attach to the edge of pre-grown MoS_2. Because of this growth mechanism, increase of grain sizes can be achieved by confining the nucleation sites, even at lower temperatures. The grain sizes of MoS_2 grown at on the different substrates were 50 (piranha treated), 70 (bare), and 100 nm (high-vacuum annealed). Confinement of the nucleation sites slightly impedes the growth time for a fully covered monolayer. The scale bar is 100 nm.

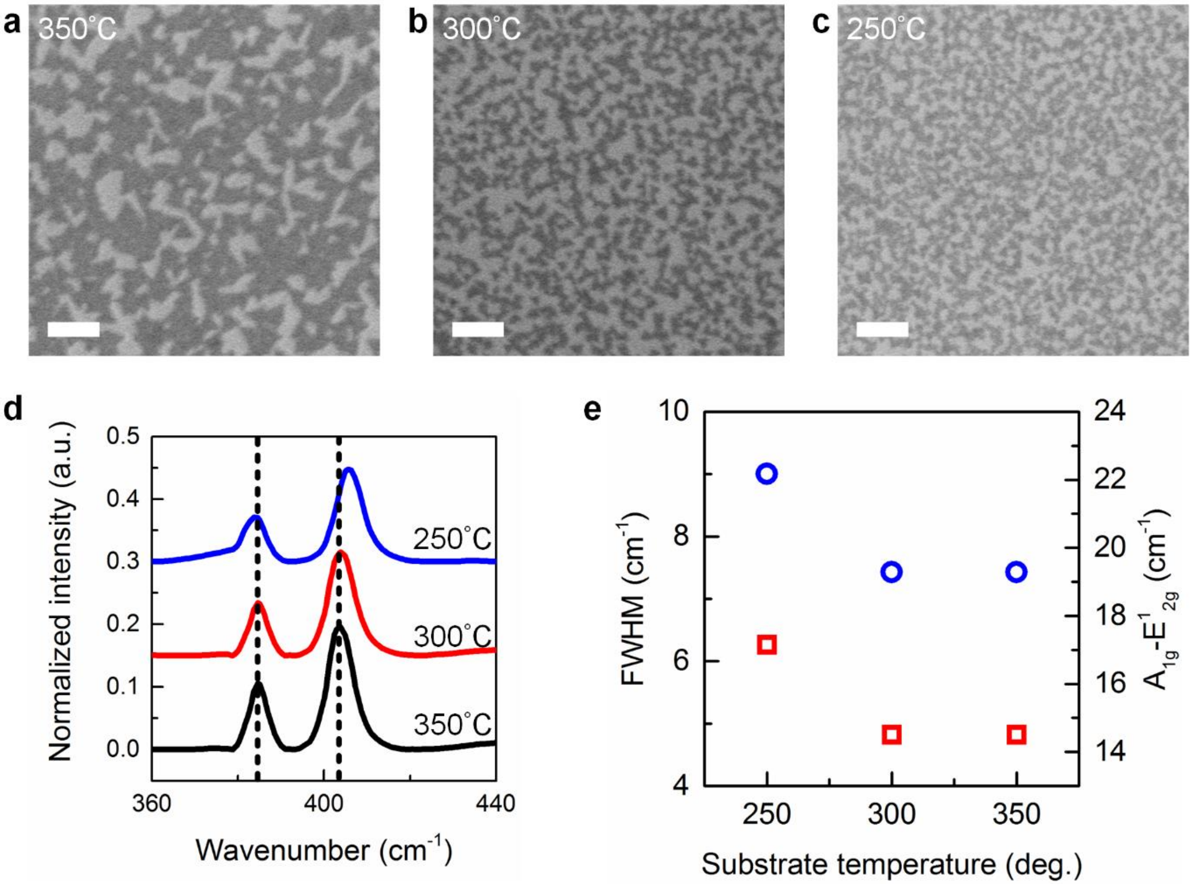

Supplementary Figure S7. Temperature effect on grain size of MoS_2.

The substrate temperature effect on MoS_2 growth was examined using SEM (Fig. S7a–c) and Raman spectroscopy measurement (Fig. S7d,e) that demonstrate the grain sizes and morphology changes by decreasing temperature. The MoS_2 grown at 250˚C showed not only smaller grain size than 350˚C case but also bilayer formation on the uncovered MoS_2 monolayer owing to short diffusion length on the surface. The scale bar is 200 nm.

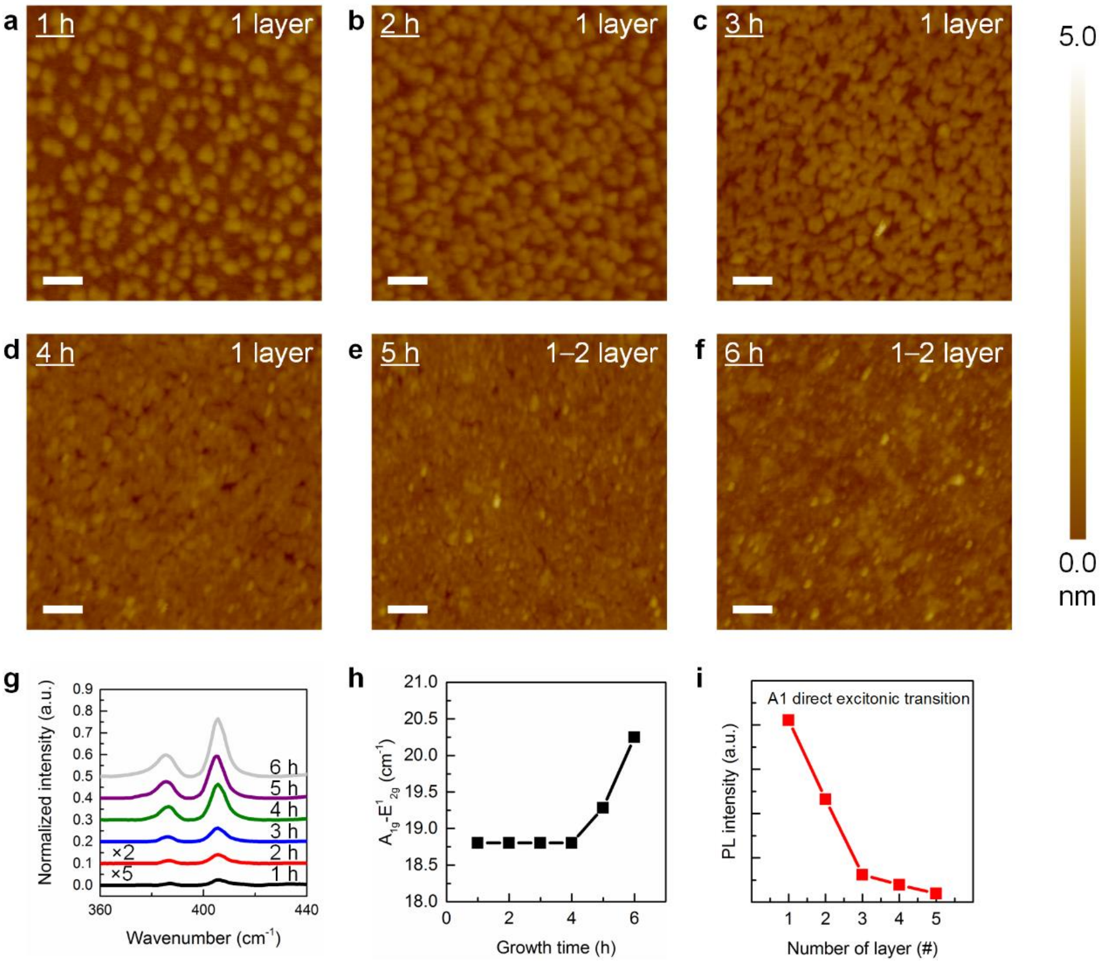

Supplementary Figure S8. Layered MoS_2 growth.

Figure S8a shows the initial phase of growth. The small-size triangular islands of monolayer MoS_2 were grown on the nucleation sites of the SiO_2 substrate. In Fig. S8b–d, the MoS_2 islands further grew and coalesced with each other to form polycrystalline monolayer MoS_2. In Fig. S8e and f, triangular MoS_2 bilayer islands were grown on fully covered monolayer. The corresponding values of Δk in the Raman spectra for each growth time were measured as 18.8, and 19.3–20.3 cm^{-1} for the monolayer and the monolayer with bilayer islands, respectively (Fig. S8g,h). In Fig. S8i, the intensity of A1 direct excitonic transition was decreased and shifted to the red with increasing number of layer. In the intermediate phase between the fully covered monolayer to bilayer, the average values of Δk were 18.8 and 22.4 cm^{-1} for the fully covered monolayer and bilayer MoS_2, respectively. The scale bar is 100 nm.

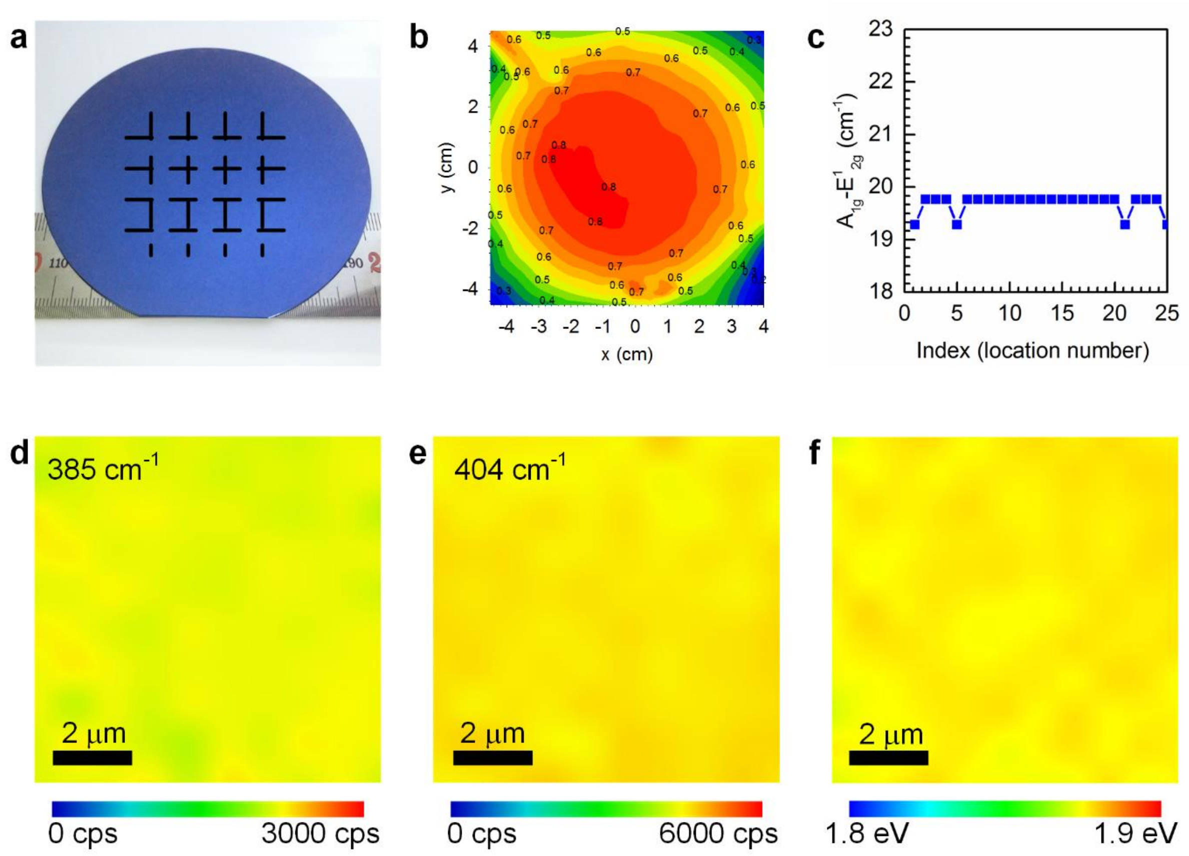

Supplementary Figure S9. Wafer-scale growth.

Figure S9a shows a photograph of monolayer MoS_2 grown on 4” SiO_2/Si wafer. The uniformity of the grown MoS_2 for entire substrate was evaluated by an ellipsometry mapping analysis (Fig. S9b) and location selected Raman spectroscopy measurement. The thickness ranged from 0.7 to 0.8 nm. MoS_2 were successfully grown over a 3” area. The local homogeneity of the central region of the wafer was also confirmed using Raman mapping (Fig. S9d,e) and PL mapping (Fig. S9f) analysis. The units of inset values in Fig. S9b are nanometers.

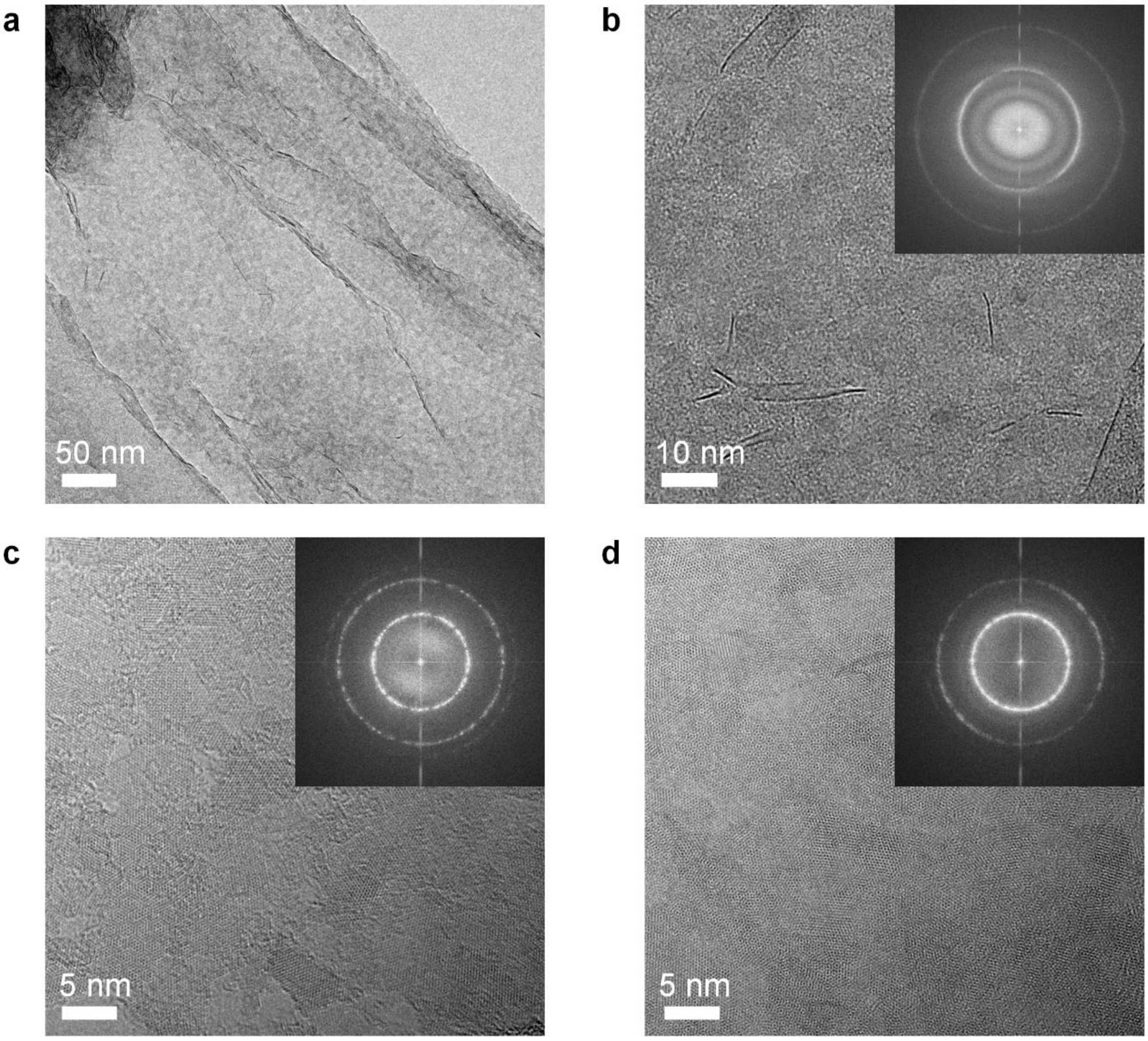

Supplementary Figure S10. Atomic structure of 3D structural MoS_2.

Figure S9a is a low-magnification HRTEM image of transferred 3D structural MoS_2 (Fig. 1a, case 1). Figure S2b–d shows the high-magnification images taken from random locations in Fig. S9a with the corresponding FFT patterns. The polycrystalline multi-layered MoS_2 consists of small-size 3D layered structural domains, and it is difficult to distinguish the grain boundaries owing to the interference of the overlapping layers and small grain sizes.

Supplementary References

1. Ambrose, D., Lawrenson, I. J. & Sprake, C. H. S. The vapour pressure of naphthalene. *J. Chem. Thermodyn.* **7,** 1173-1176 (1975).

2. Ruzicka, K., Fulem, M. & Ruzicka, V. Recommended vapor pressure of solid naphthalene. *J. Chem. Eng. Data* **50,** 1956-1970 (2005).

3. Ishikawa, Y. I. & Kawakami, K. Structure and infrared spectroscopy of group 6 transition-metal carbonyls in the gas phase: DFT studies on $M(CO)_n$ (M = Cr, Mo, and W; *n* = 6, 5, 4, and 3). *J. Phys. Chem. A* **111,** 9940-9944 (2007).

4. Windom, B. C., Sawyer, W. G. & Hahn, D. W. A Raman spectroscopic study of MoS_2 and MoO_3: Applications to tribological systems. *Tribol. Lett.* **42,** 301-310 (2011).

5. Lupan, O. et al. Investigation of optical properties and electronic transitions in bulk and nano-microribbons of molybdenum trioxide. *J. Phys. D: Appl. Phys.* **47,** 085302 (2014).

6. Helveg, S. et al. Atomic-scale structure of single-layer MoS_2 nanoclusters. *Phys. Rev. Lett.* **84,** 951-954 (2000).

7. Raybaud, P., Hafner, J., Kresse, G., Kasztelan, S. & Toulhoat, H. Ab initio study of the H_2–H_2S/MoS_2 gas–solid interface: The nature of the catalytically active sites. *J. Catal.* **189,** 129-146 (2000).

8. Lauritsen, J. V. et al. Size-dependent structure of MoS_2 nanocrystals. *Nature Nanotech.* **2,** 53-58 (2007).

9. Li, T. & Galli, G. Electronic properties of MoS_2 nanoparticles. *J. Phys. Chem. C* **111,** 16192-16196 (2007).

10. Stesmans, A. Dissociation kinetics of hydrogen-passivated P_b defects at the (111)Si/SiO_2

interface. *Phys. Rev. B.* **61,** 8393-8403 (2000).
